# Simulated weightlessness affects the expression and activity of neuronal nitric oxide synthase in the rat brain

**DOI:** 10.18632/oncotarget.15407

**Published:** 2017-02-16

**Authors:** Nara Yoon, Kiyong Na, Hyun-Soo Kim

**Affiliations:** ^1^ Department of Pathology, The Catholic University of Korea Incheon St. Mary’s Hospital, Incheon, Republic of Korea; ^2^ Department of Pathology, Severance Hospital, Yonsei University College of Medicine, Seoul, Republic of Korea

**Keywords:** simulated weightlessness, neuronal nitric oxide synthase, rat, brain, tail suspension, Pathology Section

## Abstract

Spaceflight induces pathophysiological alterations in various organs. To study pathophysiological adaptations to weightlessness on the ground, the tail suspension (TS) rat model has been used to simulate the effects of weightlessness. There is currently little information on the effect of TS on the expression and activity of nitric oxide synthase (NOS) in the brain. In this study, we examined time-dependent alterations in the expression and activity of neuronal NOS (nNOS) in the brains of TS rats. Male Sprague-Dawley rats were tail-suspended for 1 (TS1), 7 (TS7), and 14 (TS14) days or rested on the ground for 3 days after 14 days of TS. TS1 and TS7 rats exhibited no significant alterations in the expression of nNOS compared to control rats, whereas nNOS expression in TS14 rats was significantly upregulated compared to control rats. Normalized expression of nNOS mRNA and protein in TS14 rats (1.86 ± 0.48 and 1.84 ± 0.29, respectively) were significantly higher than that of control rats (*P* < 0.001 and *P* < 0.001, respectively). Consistent with these results, significant elevations in NOS activity and NO production were observed in TS14 rats. Thus, we demonstrated a significant upregulation of nNOS expression, accompanied by significant increases in NOS activity and NO production, in the brain of rats exposed to simulated weightlessness.

## INTRODUCTION

The success of space missions over the past 50 years has highlighted the necessity and advantages of human space exploration. However, as human space travel becomes more feasible in the 21st century, the health and safety of space travelers have become the most important concern. Because experiments performed in space are both demanding and expensive, several experimental models have been developed to simulate weightlessness/microgravity. Gravity is an essential force that influences many physiological functions. Indeed, changes in gravitational force affect many aspects of human pathophysiology [1]. For example, exposure to weightlessness such as that experienced by astronauts during spaceflight induces a redistribution of blood and body fluid from the caudad to cephalad portion of the body and eliminates the cephalocaudal hydrostatic pressure gradient [2]. This redistribution involves approximately 2,000 mL of blood and body fluid, leading to the so-called puffy faces and bird legs described by astronauts. In addition, astronauts frequently experience symptoms of headache and stuffiness in the nose and throat [1]. Some of these symptoms are thought to be related to changes in the cerebrospinal fluid volume and may be due to adaptation [3].

The tail suspension (TS) rat model has been used to investigate weightlessness-induced alterations including the redistribution of body fluid and volume changes in cerebrospinal fluid. The TS rat model was first introduced by Morey-Holton and was later improved by Morey-Holton and Globus [4]. The TS rat model induces a cephalic fluid shift and postural muscle unloading, which are characteristics of exposure to weightlessness [5, 6]. In addition, TS rats exhibit specific adaptations similar to those seen in astronauts during spaceflight, including postural muscle atrophy, hypovolemia, a diminished capacity for elevated vascular resistance, orthostatic hypotension, and reduced aerobic capacity [7]. Moreover, if prolonged for several days, the fluid shift that develops in TS rats alters the expression of certain genes and proteins [8].

Nitric oxide (NO) is an endogenous modulator produced by various types of cells in different tissues and regulates numerous biological processes [9]. NO is the most potent endogenous vasodilator and plays an important role in the regulation of systemic vascular resistance and tissue perfusion. In addition, NO inhibits renal tubular sodium reabsorption and contributes to sodium and extracellular fluid volume homeostasis [9–13]. Through these mechanisms, NO plays a crucial role in the regulation of blood pressure. Neuronal NO synthase (nNOS), a calcium-dependent isoform of NOS, is constitutively expressed in neuronal cells. nNOS can produce NO by converting L-arginine to L-citrulline in the presence of nicotinamide adenine dinucleotide phosphate (NADPH), oxygen, and other co-factors [14, 15]. Previous studies have shown that TS increases vascular NO activity and NOS expression in the aorta, heart, and kidneys [9, 16], suggesting that simulated weightlessness alters the expression and activity of NOS. Moreover, a few studies have documented the association between simulated weightlessness and alteration in nNOS expression or activity [9, 17]; however, there is little information about the effects of simulated weightlessness on the expression and activity of nNOS in the brain. Thus, in the present study, we examined the time-dependent alteration in nNOS expression and activity in the brain tissues of control and TS rats, an animal model of simulated weightlessness.

## RESULTS

**Figure 1:**
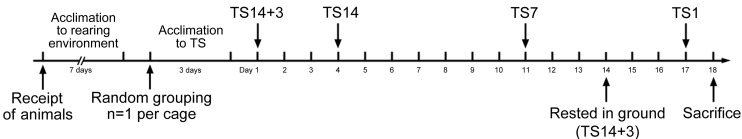
Scheme of the experimental design The experimental groups were exposed to simulated weightlessness by tail suspension (TS) for 1 (TS1), 7 (TS7), or 14 (TS14) days or rested on the ground for 3 days after 14 days of TS (TS14+3). The number of rats per group was 8.

A schematic of our experimental design is shown in Figure 1. Quantitative analysis of nNOS mRNA expression in brain tissue revealed a significant increase in TS14 rats compared to control rats (*P* < 0.001), which remained significantly high until 3 days post-suspension (TS14+3; *P* = 0.002). The maximal mRNA expression of nNOS was observed for the TS14 rats, and the increase in nNOS appeared as single peak, with TS1 and TS7 rats exhibiting a slight increase in nNOS mRNA level compared to control rats, but the differences were not statistically significant (*P* = 1.000 and *P* = 0.263, respectively). Consistent with the mRNA data, nNOS protein levels on Western blot analysis were significantly higher in the TS14 and TS14+3 rats than in the control rats (*P* < 0.001 and *P* < 0.001, respectively), while the protein expression of nNOS in the TS1 and TS7 rats was not significantly different from that of control rats (*P* = 1.000 and *P* = 1.000, respectively). The mRNA and protein expression levels of TS14+3 rats were slightly decreased compared to those of the TS14 rats, but the difference between the two groups was not statistically significant (*P* = 0.945 and *P* = 1.000, respectively). The mean nNOS expression compared between groups is shown in Table 1.

**Table 1:** Effects of simulated weightlessness by tail suspension on neuronal nitric oxide synthase expression

Group	Normalized mRNA expression ratio		Normalized protein expression ratio	
	Mean ± SEM	*P* value *vs* Control	Mean ± SEM	*P* value *vs* Control
Control	1.00		1.00	
TS1	1.23 ± 0.20	1.000	1.15 ± 0.23	1.000
TS7	1.34 ± 0.20	0.263	1.18 ± 0.15	1.000
TS14	1.86 ± 0.48	< 0.001	1.84 ± 0.29	< 0.001
TS14+3	1.61 ± 0.34	0.002	1.71 ± 0.29	< 0.001

We next measured the conversion of [^14^C]-labeled L-arginine to L-citrulline in brain tissues to quantify NOS activity. Our results revealed a concordance between NOS activity and expression: NOS activity was significantly elevated in the TS14 rats (*P* < 0.001), and this elevation persisted until 3 days post-suspension (TS14+3; *P* < 0.001). Similar to the pattern of mRNA and protein expression, the enzymatic activity of TS1 and TS7 rats revealed an increasing trend compared to the control rats, but the differences were not statistically significant (*P* = 0.238 and *P* = 0.128, respectively). The means of NOS activity for each group is shown in Table 2.

**Table 2:** Effects of simulated weightlessness by tail suspension on nitric oxide synthase activity nitric oxide production

Group	NOS activity (pM/mol/mg)		Nitrate plus nitrite level (μM/mg)	
	Mean ± SEM	*P* value *vs* Control	Mean ± SEM	*P* value *vs* Control
Control	0.08 ± 0.04		12.03 ± 3.26	
TS1	0.12 ± 0.02	0.238	12.35 ± 4.81	1.000
TS7	0.13 ± 0.02	0.128	15.58 ± 1.91	1.000
TS14	0.26 ± 0.04	< 0.001	30.60 ± 7.35	< 0.001
TS14+3	0.17 ± 0.05	< 0.001	23.59 ± 7.45	0.001

Level of nitrate plus nitrite in the brain exhibited a trend similar to that of NOS activity and expression. Specifically, nitrate plus nitrite levels were significantly elevated in TS14 rats (*P* < 0.001), remained significantly high through 3 days post-suspension (TS14+3; *P* < 0.001), and the difference in levels between the two groups was not statistically significant (*P* = 0.140). Lastly, the TS1 and TS7 rats exhibited similar NO production to that of the control rats (*P* = 1.000 and *P* = 1.000, respectively). The mean level of nitrate plus nitrite level for each group is shown in Table 2.

**Figure 2:**
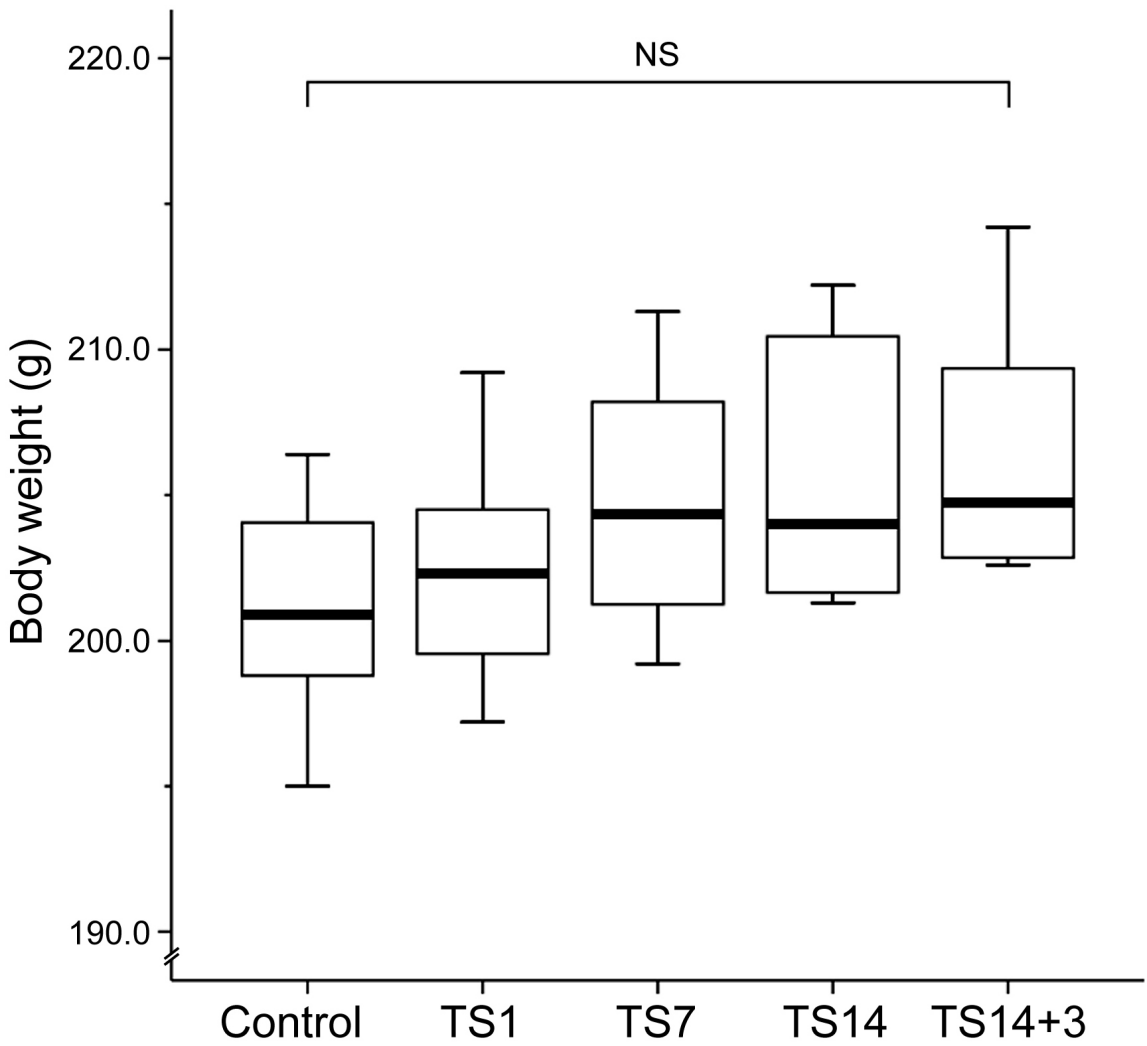
Effects of simulated weightlessness by tail suspension on body weight Box and whisker diagram. The band inside each box is the median value of the indicated group. Body weight exhibited an increasing trend during the observation period, but the differences among groups were not statistically significant.

Previous studies stated that TS does not appear to be stressful to animals, as assessed by corticosterone level, weights of adrenal glands and thymus, and total body weight [18, 19]. In those studies, food and water intake, grooming, defecation, and urination behavior were used as indications that the rats were not under overt stress. In addition, the animals that exhibited excessive weight loss (more than 10%) or overt signs of stress were removed from the study [20]. In the present study, no rats showed weight loss. We observed that the TS rats had higher body weights than the control rats (Figure 2), although there were no significant differences in body weight between the controls (201.11 ± 1.36 g) and the rats in the TS1 (202.39 ± 1.37 g; *P* = 1.000), TS7 (204.76 ± 1.52 g; *P* = 0.928), TS14 (205.71 ± 1.64 g; *P* = 0.362), or TS14+3 (206.34 ± 1.55 g; *P* = 0.184). Thus, on the basis of the above-mentioned criteria, we considered that the TS model employed in the present study did not induce overt chronic stress.

**Figure 3:**
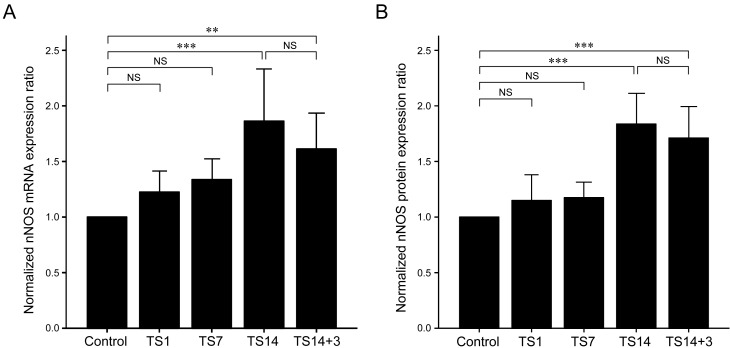
Effects of simulated weightlessness by tail suspension (TS) on neuronal nitric oxide synthase (nNOS) expression **A**. nNOS mRNA expression was significantly elevated at 14 days of TS (*P* < 0.001) and remained significantly high after 3 days post-suspension (*P* = 0.002). **B**. Significantly elevated nNOS protein levels were observed in the TS14 (*P* < 0.001) and TS14+3 (*P* < 0.001) rats. The expression levels of both mRNA and protein were slightly decreased in the TS14+3 rats compared to those in the TS14 rats, but the differences between the two groups were not statistically significant.

**Figure 4:**
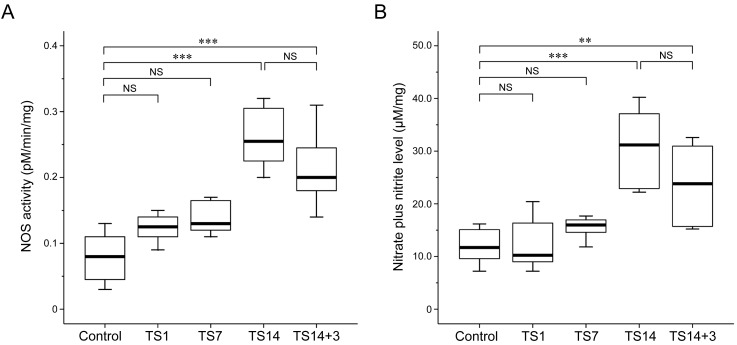
Effects of simulated weightlessness by tail suspension (TS) on nitric oxide synthase (NOS) activity and nitric oxide (NO) production Data are presented as box and whisker diagrams, and the band inside each box is the median value of the indicated group. An L-[^14^C]-citrulline assay and a nitrate plus nitrite assay revealed similar trends with respect to the levels of NOS enzymatic activity and NO production, both of which were significantly increased after 14 days of TS. **A**. NOS activity was significantly upregulated in TS14 (*P* < 0.001) and TS14+3 (*P* < 0.001) rats. **B**. The maximal nitrate plus nitrite level was observed after 14 days of TS. The time-dependent alteration pattern was consistent with that of NOS expression and activity.

## DISCUSSION

There have been relatively few studies on the effects of simulated weightlessness on the brain. A study using cDNA microarrays showed that TS induces considerable transcriptomic alterations in several functional pathways including those involving blood coagulation, immune responses, and ion channels [1]. Proteomic analysis in the hippocampus of TS mice revealed changes in structural proteins coupled with the loss of proteins involved in cellular metabolism [21]. A similar analysis performed in the hypothalamus revealed alteration in biomarkers of oxidative stress, indicating vulnerability of the hypothalamus to the stress generated by weightlessness [22]. Taken together, these studies suggest that exposure to weightlessness affects several aspects of brain function. Meanwhile, there have been only two studies to date that have investigated the effects of simulated weightlessness on the expression of nNOS in the brain. Mueller et al. [17] showed a higher nNOS protein expression in the paraventricular and supraoptic nuclei of the hypothalamus of rats subjected to TS for 14 days. Similarly, Vaziri et al. [9] observed a significant elevation of nNOS protein expression in the brains of rats exposed to simulated weightlessness by TS for 20 days. Taken together, these data suggest that increased nNOS in the brain is involved in the regulation of vasodilatory tone and central sympathetic outflow and contributes to autonomic and humoral alterations following cardiovascular deconditioning.

Consistent with previous data, the TS rats employed in this study exhibited a significant upregulation of brain nNOS expression and activity. nNOS is normally expressed in various regions of the brain and is thought to be involved in neurogenic control of blood pressure by inhibiting central sympathetic outflow [9, 23–25]. Consequently, nNOS-derived NO in the brain is considered to exert a blood pressure-lowering influence. Our observations raise the possibility that upregulation of brain nNOS may contribute to orthostatic intolerance by suppressing central sympathetic outflow following exposure to weightlessness. In support of this notion, Moffitt et al. [26] reported a marked attenuation of sympathetic nerve activity in TS rats compared to control rats. Moreover, previous studies using several animal models of hypertension have demonstrated that NOS-derived NO modulates sympathetic nervous activity. Ye et al. [27] observed that treatment with L-arginine, the substrate of NOS, attenuates the severity of hypertension in rats with chronic renal failure, whereas treatment with L-N^G^-nitroarginine methyl ester (L-NAME), a competitive inhibitor of NOS, causes accelerated hypertension. In that study, the authors also noted a dose-response relationship between the amount of L-NAME ingested and the severity of hypertension as well as a positive relationship between NOS expression and norepinephrine turnover rate after treatment with L-N^G^-nitroarginine methyl ester, suggesting that endogenous NO inhibits sympathetic nervous activity in the brain and is involved in the neurogenic inhibitory regulation of blood pressure. Ni et al. [28] observed that a high-salt diet upregulated nNOS expression in the brain, suggesting the involvement of NOS in the control of blood pressure. These data also suggest a possible role of elevated blood pressure in the regulation of brain nNOS expression. A previous study showed that shifts in gravitational force markedly raised intracranial blood pressure, although TS rats did not develop systemic hypertension [5, 29]. This local rise in blood pressure in the cerebral circulation may be responsible for the observed upregulation of brain nNOS expression in TS rats.

Although quantitative real-time reverse transcriptase-polymerase chain reaction (RT-PCR) and Western blot analysis demonstrated a significant upregulation in nNOS expression in the given tissues, it did not identify the specific regions or cell types that contributed to this phenomenon. However, based on previous observations by Mueller et al. [17], the source of increased nNOS expression in the brain of TS rats appears to be the hypothalamus, especially the paraventricular and supraoptic nuclei. Immunohistochemical localization of nNOS in sequential brain tissue samples obtained from TS rats will be necessary to confirm the time-dependent alteration in nNOS expression observed in this study.

In conclusion, exposure of rats to simulated weightlessness in the TS model resulted in a significant increase in brain nNOS expression. This upregulation of nNOS expression in TS rats was coupled with significant increases in NOS activity and NO production. Our observations raise the possibility that upregulation of brain nNOS may contribute to orthostatic intolerance by suppressing central sympathetic outflow following exposure to weightlessness.

## MATERIALS AND METHODS

### Tail suspension rat model

This study used male Sprague-Dawley rats eight weeks of age and weighing 190‒210 g. Rats were fed standard laboratory rat chow, provided free access to water, and were maintained on a 12-hour light/dark cycle under temperature and moisture levels controlled at 20‒25°C and 40‒45%, respectively. To avoid any effect of unfavorable factors including fear and stress, the rats were acclimatized to the rearing environment for 7 days before the beginning of experiments. Animals were randomly assigned to one of four experimental groups or one control group. Rats in the experimental groups were tail-suspended for 1 (TS1; n=8), 7 (TS7; n=8), or 14 (TS14; n=8) days or rested on the ground for 3 days after 14 days of TS (TS14+3; n=8). The TS rat model was established as described previously [30, 31]. Briefly, the rats underwent an acclimation period during which they were tail-suspended for short durations (3 days; 1–3 hours per day) by either tail harness or stainless steel rings. For suspension, the tail was cleaned and dried, and adhesive sponge tape strips were adhered laterally along the two sides of the proximal two-thirds of the tail. These longitudinal strips were then secured to the tail by three 1-cm-wide tape strips wrapped circumferentially at three sites along the length of the tail. To facilitate free movement about the cage, the cast was attached to a swivel anchored to the cage top, allowing a 360° range of movement. The rats were caged individually and maintained in a head-down tilt position of approximately 30°, with their hindlimbs unloaded. The control rats (n=8) in individual cages were treated identically except for the TS. During TS, the angle of suspension was re-adjusted as body size increased to prevent weight bearing on the hindlimbs. Because the TS rats had limited movement, they were groomed daily to prevent complications such as urine scald and infection. Daily health checks were performed to confirm that the exposed tip of the tail remained pink, which was taken as a sign of adequate blood flow.

After the TS period, rats were anesthetized with 100% carbon dioxide for 90 seconds. The whole brain was removed, cleaned in phosphate buffered saline, snap frozen in liquid nitrogen, and stored at –70°C until processed. All experiments were approved by the Institutional Animal Care and Use Committee of the Republic of Korea Air Force Aerospace Medical Center (Cheongju, Chungcheongbuk-do, Republic of Korea). All experimental procedures involving animals were conducted in accordance with the Guide for Care and Use of Laboratory Animals published by the National Institutes of Health and the ethical guidelines of the International Association for the Study of Pain.

### Quantitative real-time reverse transcriptase-polymerase chain reaction analysis

Total RNA was isolated using the NucleoSpin RNA II extraction kit (Macherey-Nagel GmbH & Co. KG, Dueren, Germany) and used for cDNA synthesis with a ReverTra Ace-α- reverse transcriptase kit (Toyobo Co., Ltd., Osaka, Japan); both kits were used according to the manufacturers’ instructions. The amount of cDNA was determined photometrically. RT-PCR was performed using cDNA and a SsoAdvanced SYBR Green Supermix (Bio-Rad Laboratories, Inc., Hercules, CA, USA). PCR was performed using a Bio-Rad CFX96 Real-Time PCR Detection System (Bio-Rad Laboratories, Inc.) with a C1000 Thermal Cycler (Bio-Rad Laboratories, Inc.). PCR reactions for nNOS and glyceraldehyde 3-phosphate dehydrogenase (GAPDH) were initiated with a denaturing step at 95°C for 3 minutes, followed by 40 cycles at 95°C for 10 seconds, 58°C for 10 seconds, and 72°C for 20 seconds. A melting curve (ramping from 65–95°C) was performed following RT-PCR to test for the presence of primer dimers. The primer sequences for nNOS were 5′-GGC ACT GGC ATC GCA CCC TT-3′ (sense) and 5′-CTT TGG CCT GTC CGG TTC CC-3′ (antisense). The primer sequences for GAPDH were 5′-CAA GAA GGT GGT GAA GCA-3′ (sense) and 5′-GGT GGA GAG TGG GAG TT-3′ (antisense). In cases where a primer dimer was detected, PCR was repeated using a separate cDNA aliquot. Each measurement was repeated three times, and the values were used to calculate the ratio of nNOS to GAPDH, with the control set at a value of 1.0 to serve as a standard.

### Western blot analysis

Tissue samples were homogenized in lysis buffer (50 mM Tris, pH 7.5, 1% Nonidet P-40, 2 mM ethylenediaminetetraacetic acid, 10 mM sodium chloride, 20 μg/mL aprotinin, 20 μg/mL leupeptin, and 1 mM phenylmethylsulfonyl fluoride) at a volume of 1 mL per 100 mg tissue and placed on ice for 20 min. After centrifugation at 13,000 rpm for 20 min, the supernatant was collected and used for immunoblotting [32, 33]. Immunoblots were incubated with anti-nNOS antibody (1:500; Novus Biologicals, LLC, Littleton, CO, USA) or anti-GAPDH antibody (1:800; Novus Biologicals, LLC), followed by incubation with horseradish peroxidase-conjugated secondary antibody (Cell Signaling Technology, Beverly, MA, USA). Protein bands were visualized using an enhanced chemiluminescence reagent (Amersham Biosciences, Buckinghamshire, UK) according to the manufacturer's instructions.

### Measurement of nitric oxide synthase activity with the L-[14C]-citrulline formation assay

Tissue samples were ground to a fine powder in liquid nitrogen and homogenized in a solution containing 10 mM 4-(2-hydroxyethyl)-1-piperazineethanesulfonic acid (pH 7.2), 0.32 M sucrose, 0.1 mM ethylenediaminetetraacetic acid, 1 mM dithiothreitol, and protease inhibitors (10 μg/mL each of leupeptin, pepstatin A, chymostatin, antipain, and soybean trypsin inhibitor and 100 μg/mL phenylmethylsulfonyl fluoride) [32, 33]. To differentiate calcium-dependent NOS activity from calcium-independent NOS activity, homogenates were centrifuged, and the supernatants were incubated at 37°C for 60 min with one of the following mixtures: (1) assay cocktail: 2 μM L-[^14^C]-arginine (General Electric Healthcare, Milwaukee, WI, USA), 18 μM L-arginine, 1 mM L-citrulline, 50 mM L-valine, 1 mM dithiothreitol, 0.1 mM NADPH, 0.1 mM tetrahydrobiopterin, 1 mM magnesium chloride, 0.2 mM calcium chloride, and 50 mM potassium hydrogen phosphate, pH 7.2 (Sigma-Aldrich, St. Louis, MO, USA); (2) cocktail with 1 mM ethylene glycol tetraacetic acid; or (3) cocktail with 1 mM ethylene glycol tetraacetic acid plus 1 mM L-N^G^-monomethyl-L-arginine, monoacetate salt. NOS activity was quantified by measuring L-[^14^C]-citrulline with a liquid-scintillation counter (Wallac 1410, Pharmacia LKB Biotechnology AB, Uppsala, Sweden), following removal of unreacted L-[^14^C]-arginine with Dowex 50W-X8 cation-exchange resin (Sigma-Aldrich).

### Measurement of nitric oxide production with nitrate plus nitrite assay

NO production was indirectly quantified by measuring the amount of nitrate plus nitrite in supernatants from tissue homogenates using a Nitrate/Nitrite Colorimetric Assay Kit (Cayman Chemicals, Ann Arbor, MI, USA) according to the manufacturer's instructions [32, 33]. Briefly, 5-μL aliquots were injected into a Sievers Nitric Oxide Analyzer (NOA 280i; Sievers Instruments, Boulder, CO, USA) and pelleted by centrifugation with acetic acid as a reductant for nitrite and vanadium chloride and hydrogen chloride as reductants for nitrate and sodium iodide, respectively. Values were normalized for protein concentration as determined using the Bradford reagent. Nitrate plus nitrite level was expressed in units of μM based on tissue weight.

### Statistical analysis

All values are provided as the mean ± standard error. Differences in the normalized mRNA and protein expression ratio, enzymatic activity, and nitrite plus nitrate concentration between the groups were assessed using one-way analysis of variance (ANOVA), followed by Bonferroni post hoc test. Statistical analyses were conducted using PASW Statistics for Windows (version 18.0; Armonk, NY, USA). Statistical significance was defined as a P value less than 0.05.
